# Commercially available artificial intelligence tools for fracture detection: the evidence

**DOI:** 10.1093/bjro/tzad005

**Published:** 2023-12-12

**Authors:** Cato Pauling, Baris Kanber, Owen J Arthurs, Susan C Shelmerdine

**Affiliations:** UCL Great Ormond Street Institute of Child Health, University College London, London WC1E 6BT, United Kingdom; Queen Square Multiple Sclerosis Centre, Department of Neuroinflammation, University College London (UCL) Queen Square Institute of Neurology, Faculty of Brain Sciences, University College London, London WC1N 3BG, United Kingdom; Department of Medical Physics and Biomedical Engineering, Centre for Medical Image Computing, University College London, London WC1E 6BT, United Kingdom; UCL Great Ormond Street Institute of Child Health, University College London, London WC1E 6BT, United Kingdom; Department of Clinical Radiology, Great Ormond Street Hospital for Children NHS Foundation Trust, London WC1N 3JH, United Kingdom; NIHR Great Ormond Street Hospital Biomedical Research Centre, Bloomsbury, London WC1N 1EH, United Kingdom; UCL Great Ormond Street Institute of Child Health, University College London, London WC1E 6BT, United Kingdom; Department of Clinical Radiology, Great Ormond Street Hospital for Children NHS Foundation Trust, London WC1N 3JH, United Kingdom; NIHR Great Ormond Street Hospital Biomedical Research Centre, Bloomsbury, London WC1N 1EH, United Kingdom

**Keywords:** machine learning, artificial intelligence, fracture, commercial, radiology, imaging

## Abstract

Missed fractures are a costly healthcare issue, not only negatively impacting patient lives, leading to potential long-term disability and time off work, but also responsible for high medicolegal disbursements that could otherwise be used to improve other healthcare services. When fractures are overlooked in children, they are particularly concerning as opportunities for safeguarding may be missed. Assistance from artificial intelligence (AI) in interpreting medical images may offer a possible solution for improving patient care, and several commercial AI tools are now available for radiology workflow implementation. However, information regarding their development, evidence for performance and validation as well as the intended target population is not always clear, but vital when evaluating a potential AI solution for implementation. In this article, we review the range of available products utilizing AI for fracture detection (in both adults and children) and summarize the evidence, or lack thereof, behind their performance. This will allow others to make better informed decisions when deciding which product to procure for their specific clinical requirements.

## Introduction

Missed fractures impact both patients and healthcare providers. Between 2015 and 2018, the total cost of missed fracture claims in the NHS was over £1.1 million, with an average cost per claim of approximately £14 000.^1^ Although the number of claims were relatively few (*n* = 78) compared to over 1 million fracture attendances per year across the United Kingdom, they are seen as an avoidable cost, money which could be better spent improving other NHS services. Furthermore, missed fractures in young children pose an additional challenge, as these may reflect failed opportunities for safeguarding and referral to social services.

One means to reduce such errors may be to incorporate assistance from artificial intelligence (AI). Several systematic reviews and meta-analyses have investigated the use of AI for the detection of fractures. In adults, high sensitivity rates of 92% for plain radiographs and 89% for computerised tomography (CT) scans have been reported, with specificities of 91% for plain radiographs and 92% for CT scans^2–4^; with accuracy rates of between 89% and 98% in children.^5^ Despite such promising results, over half of these studies had a high risk of bias,^2^ were only used in a research setting, were not ready for clinical deployment, nor externally validated.^6^ This is particularly concerning given that 24% of AI solutions in one study yielded a substantial decline in performance when evaluated on external data (compared to their internal data set) and the majority (81% of algorithms) reported negative impact.^7^

**Figure 1.**
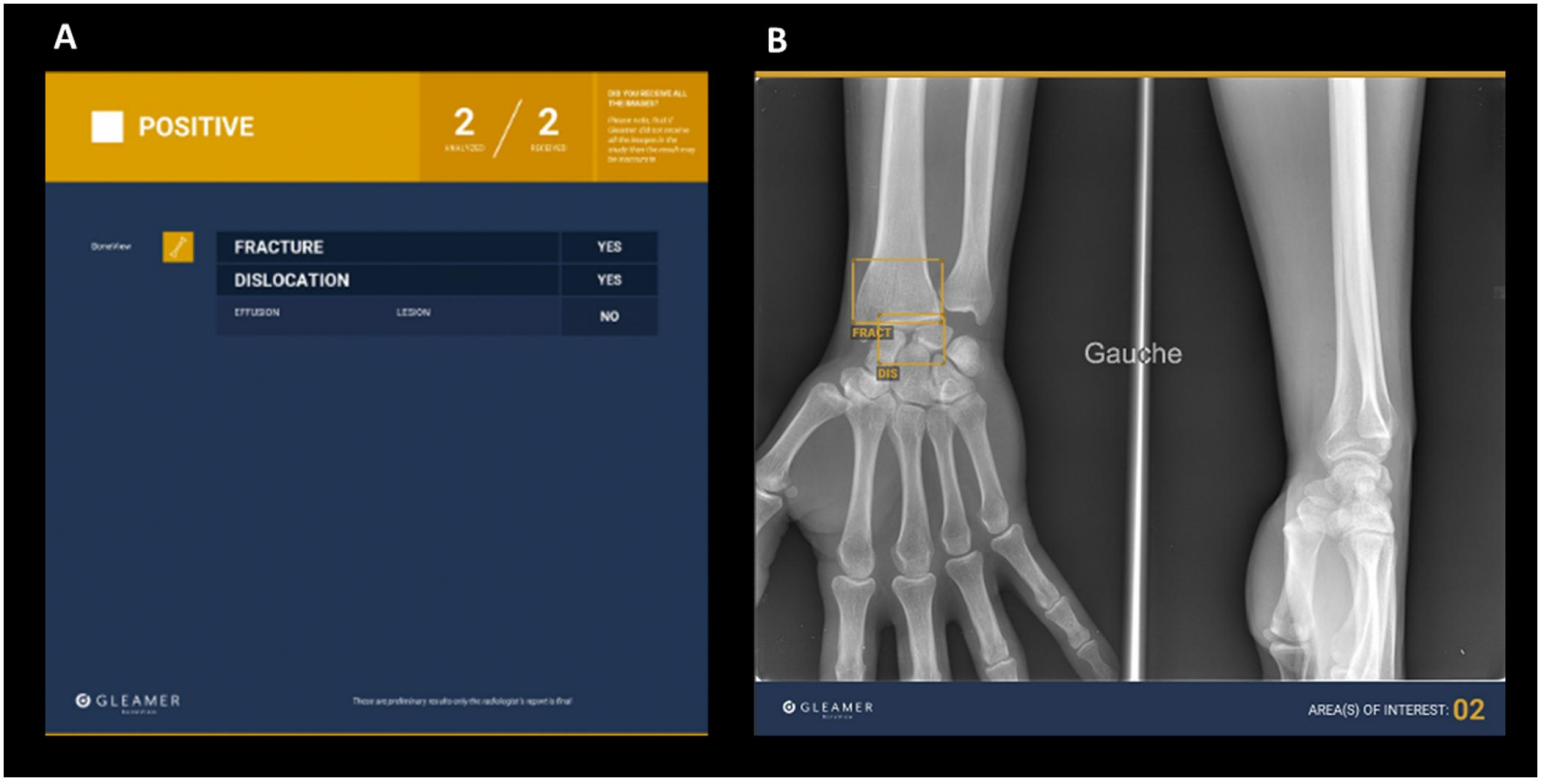
This image demonstrates the results produced when using an artificial intelligence (AI) fracture detection tool by Gleamer, called BoneView. With this product, a summary image is sent to PACS, depicted in figure (A) which shows the number and type of pathologies detected on the radiograph. (B) A second image is also sent to PACS with bounding boxes and their associated labels (FRACT = fracture, DIS = dislocation) displayed as an “overlay” across the original radiographic image in question. In this example, the AI has flagged a fracture of the distal radius, with a scapholunate dislocation in a child. Image provided by Daniel Jones, Gleamer.

**Figure 2.**
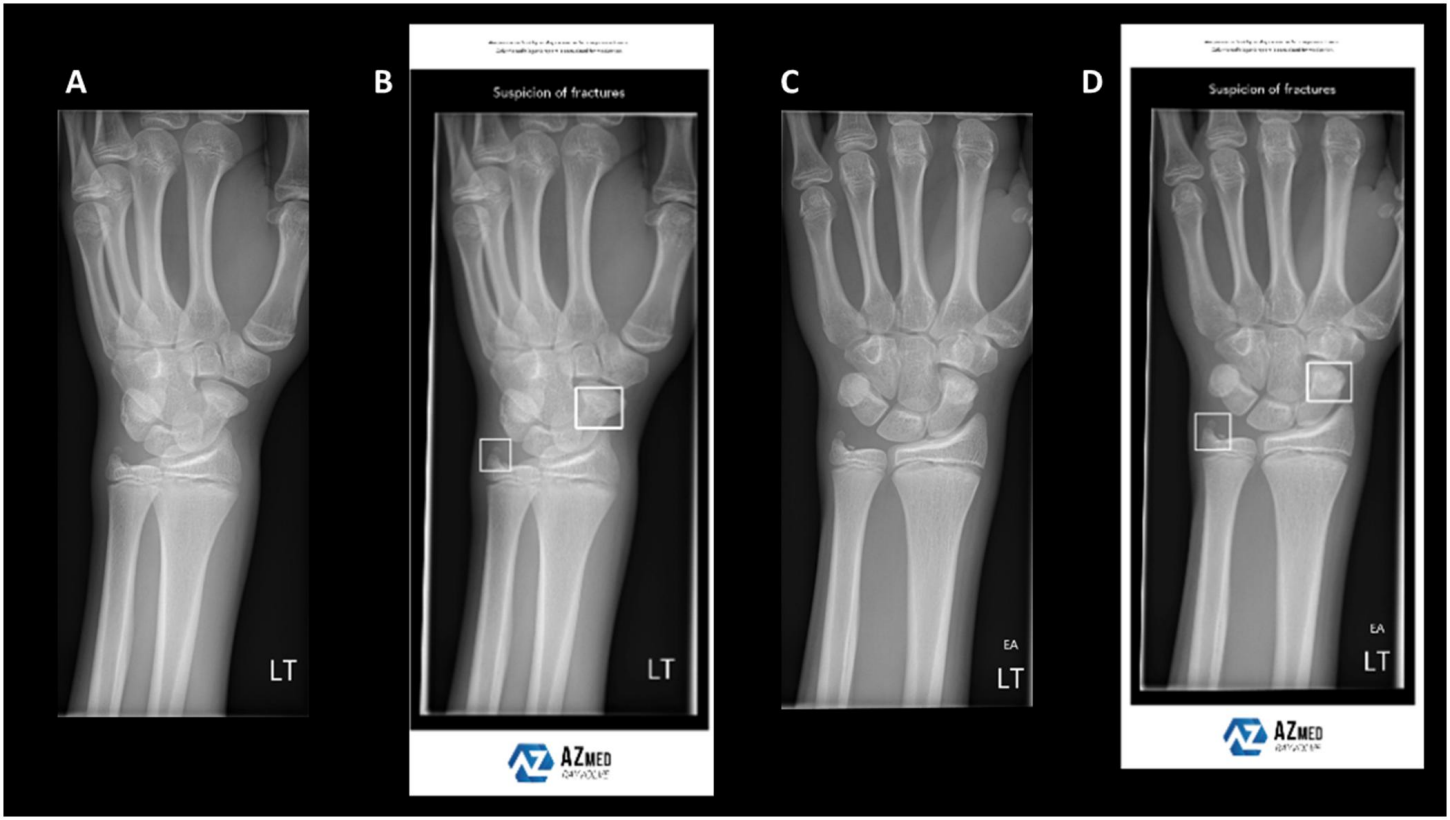
This image demonstrates the results produced when using an artificial intelligence (AI) fracture detection tool by AZMed, called Rayvolve. In this example, an oblique left wrist view (A) and DP wrist view (C) have been submitted for AI interpretation. The AI has flagged a fracture of the scaphoid and ulnar styloid (B, D) in a child by displaying bounding boxes as an “overlay” across the respective radiographic images. These are also sent to PACS for radiology reporter and clinician review. Image provided by Liza Alem, AZmed.

**Figure 3.**
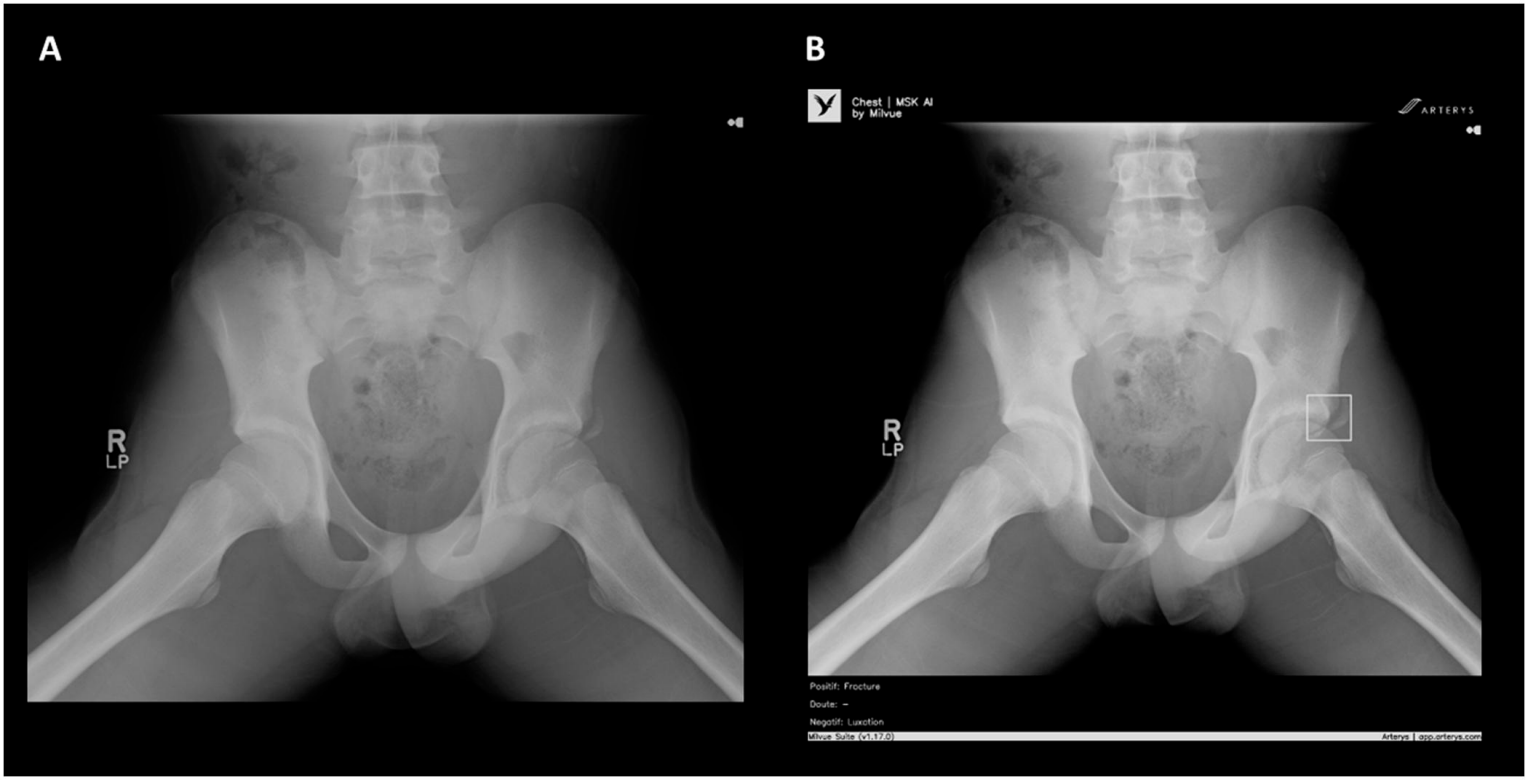
This image demonstrates an example of results produced when using an artificial intelligence (AI) fracture detection tool by Milvue, called Smarturgences. In this example, frog leg view of the pelvis in a child has been submitted for AI interpretation (A). The AI has correctly identified a fracture of the left anterior inferior iliac spine and placed a bounding box around the abnormality, as well as stating the pathology below the image (B).

Nonetheless, several AI vendors have now developed fracture detection models for routine practice (Figures 1–3), ready for commercial integration into radiological workflows. Although this brings cutting-edge technology a step closer to direct patient benefit, it is vital that such tools are independently evaluated prior to adoption. Of concern, van Leeuwen et al^8^ found that in a review of 100 Conformité Européenne (CE) marked AI tools for different radiology use cases, only 36% had any associated peer reviewed verification of performance, of which fewer than half were independent of the vendor (ie, without flagrant conflict of interest). Without independent evidence, it can be very challenging to know how well an AI product might perform in a given clinical setting, and whether it is “worth” purchasing.^9^

In this market research review, we assess the range of available commercial products utilizing AI for fracture detection (in both adults and children) and summarize the evidence behind the performance of these tools. This will allow others to make better informed decisions when deciding which AI product, if any, to procure for their specific clinical requirements.

## Methods

A search of commercially available AI solutions for fracture detection using medical images was performed by the lead and senior author, using the methods detailed below to ensure as comprehensive a search as possible.

1. A search of both CE^8^^,^^10^ certified and Food and Drug Administration (FDA) approved sites^11^^,^^12^ were filtered for medical products that utilized machine learning (ML) algorithms, and then further refined to incorporate those that included the term “fractures” within the diseases targeted by the product.
2. A review of AI exhibitors and sponsors at several large annual radiology conferences in 2022 were identified relating to general, paediatric, and musculoskeletal imaging (ie, Radiological Society of North America—RSNA, European Congress of Radiology—ECR, European Society of Paediatric Radiology—ESPR, European Society for Skeletal Radiology—ESSR) to determine if they provided solutions for fracture detection.
3. A search of peer reviewed publications within the PubMed, Scopus, and EMBASE databases published between January 1, 2012 and December 31, 2022 using a Boolean search strategy to find those articles with keywords relating to “machine learning,” “artificial intelligence,” “imaging,” and “fracture.” This was then further manually reviewed for specific mention and evaluation of commercial AI solutions.

There were no restrictions placed on the type of imaging modality, body parts targeted, type of AI/ML methodology or the intended population for the AI tool. Products and applications returned through these search strategies were deemed eligible if they supported healthcare professionals at image diagnosis, triage, classification/detection, with respect to fracture detection. We excluded products that were advertised via software marketplace redistributions, and those that were categorized under medical image management and processing systems. Only software that was currently commercially available was included in the main analysis. Products which were still in development or at prototype stage were included separately in case of future relevance to readers. Applications that have been withdrawn from the market or no longer available were not included.

For each product, information was gathered through numerous sources. Company websites, FDA/CE certification documents, user manuals, and scientific articles were first collated to collect data about the developer, technical specifications, specific functionalities relating to clinical application, and any evidence that exists to support the product’s performance. Levels of evidence for each AI solution were further classified into 6 levels according to an adapted hierarchical model of efficacy by Fryback and Thornbury,^13^ and used in a prior article evaluating evidence for commercial AI products (Table 1).^8^

**Table 1.** Hierarchical model of efficacy to assess the contribution of AI software to the diagnostic imaging process, reproduced from van Leeuwen et al,^8^ originally adapted from Fryback and Thrornbury (1991)^13^ under the Creative Commons Attribution 4.0 International Licence.^14^

Level	Explanation	Typical measures
Level 1t	Technical efficacy Article demonstrates the technical feasibility of the software	Reproducibility, inter-software agreement, error rate
Level 1c	Potential clinical efficacy Article demonstrates the feasibility of the software to be clinically applied	Correlation to alternative methods, potential predictive value, biomarker studies
Level 2	Diagnostic accuracy efficacy Article demonstrates the stand-alone performance of the software	Standalone sensitivity, specificity, area under the ROC curve, or Dice score
Level 3	Diagnostic thinking efficacy Article demonstrates the added value to the diagnosis	Radiologist performance with/without AI, change in radiological judgement
Level 4	Therapeutic efficacy Article demonstrates the impact of the software on the patient management decisions	Effect on treatment or follow-up examinations
Level 5	Patient outcome efficacy Article demonstrates the impact of the software on patient outcomes	Effect on quality of life, morbidity, or survival
Level 6	Societal efficacy Article demonstrates the impact of the software on society by performing an economic analysis	Effect on costs and quality-adjusted life years, incremental costs per quality-adjusted life year

Abbreviations: Level 1t = level 1, technical, Level 1c = level 1, clinical.

To ensure accuracy, timeliness and comprehensiveness of online information, all relevant AI vendors were contacted directly and further supplied a survey of questions (see Supplementary Material) to complete. A timeframe of 2 weeks was provided for return of the survey, with the option of having follow-up emails and online meetings to better discuss our survey queries, if preferred. We also contacted the MHRA directly with the vendor names and AI solutions to confirm whether United Kingdom Conformity Assessed (UKCA) certification had been awarded for the products in question.

## Results

In total, 21 commercial AI products across 15 different AI vendors (Tables 2-5) were identified, with a further 3 products across 3 companies at prototype/pre-market stage. These prototypes comprised 2 products which could detect fractures on radiographs (Fraxpert, SeeAI,^54^ Imera-MSK, Imera.ai^55^) and one for rib fractures on CT imaging (Dr.Wise@ChestFracture v1.0, no vendor name identified^56^).

**Table 2.** Use cases and the intended population for commercial AI tools for fracture detection on medical imaging.

Company	Product, version	Modality	Disease(s) targeted	Fracture type	Notable inclusions (for fractures)	Notable exclusions (for fractures)	Target population
Gleamer	BoneView 1.1-US	Radiography	Bone fractures	Acute and healing	Ankle, foot, knee, tibia/fibula, wrist, hand, elbow, forearm, humerus, shoulder, clavicle, pelvis, hip, femur, ribs, thoracic spine, and lumbosacral spine	Cervical spine and skull radiographs	Adults (>21 years) for all body parts and children/adolescents (2-21 years) for all body parts except pelvis, hip, femur, ribs, thoracic spine, and lumbosacral spine
Radiobotics	RBfracture v.1	Radiography	Bone fractures	Acute and healing	Appendicular skeleton only	Spine, rib, and craniofacial fractures	Vendor states product is intended for adult and paediatric use (>2 years)
AZmed	Rayvolve v2.5.0	Radiography	Traumatic injuries (fractures, dislocations, joint effusions) and chest pathologies (pneumothoraces, cardiomegaly, pleural effusions, pulmonary oedema, consolidation, nodules)	Acute and healing	–	Dental, facial, skull, and spineradiographs.	Adult only according to FDA clearance, however, evidence for performance in children has been conducted
Rayscape	Chest X-ray	Radiography	17 classes of pathologies on chest radiographs, of which one is fractures (others include pulmonary- and cardiac-related findings, as well as scoliosis)	Acute	Only frontal AP or PA chest radiographs	Not lateral chest X-rays	Adults (>16 years old)
Milvue	SmartUrgences	Radiography	7 pathologies, including bone fractures, joint effusions, joint dislocations (for musculoskeletal radiographs) and pleural effusions, pneumothorax, pulmonary opacification, pulmonary nodules (on chest radiographs)	Acute	–	Excludes axial skeleton, dental, and abdominal radiographs	Vendor states product is intended for adult and paediatric use (lower age limit not defined)
Imagen Technologies	OsteoDetect	Radiography	Wrist fractures	Acute	Distal radial fractures only	–	Adults (>22 years)
Imagen Technologies	FractureDetect	Radiography	Fractures (upper/lower extremities)	Acute	Only includes ankle (frontal, lateral, oblique), clavicle (frontal), elbow (frontal, lateral), femur (frontal, lateral), forearm (frontal, lateral), hip (frontal, frog leg lateral), humerus (frontal, lateral), knee (frontal, lateral), pelvis (frontal), shoulder (frontal, lateral, axillary), tibia/fibula (frontal, lateral), wrist (frontal, lateral, oblique)		Adults (>22 years)
Annalise.AI	Annalise Enterprise CXR v1.2	Radiography	124 pathologies on chest radiographs (including fractures and bone lesions)	Acute and healing	Clavicle, spine, ribs, humerus, scapula	–	Adults >16 years
Deepnoid	DEEP: SPINE-CF-01	Radiography and MRI	Major spinal abnormalities such as compression fractures, scoliosis angles, intervertebral disc abnormalities	Acute	Spine only	–	Adult (>20 years)
Quibim	Chest X-Ray Classifier	Radiography	Atelectasis, cardiomegaly, consolidation, oedema, emphysema, enlarged cardiomediastinum, fibrosis, fracture, hernia, lung lesion, lung opacity, pleural effusion, pleural thickening, pneumothorax	Acute	Rib fractures	–	Age not specified, presumed only adults
SenseTime	SenseCare-Chest DR Pro	Radiography	Pneumonia, tuberculosis, pneumothorax, pleural effusion, cardiomegaly, rib fractures	Acute	Rib fractures	–	Age not specified, presumed only adults
Infervision	InferRead DR Chest v1.0.1.1	Radiography	Lung cancer, pneumothorax, fracture, tuberculosis, lung infection, aortic calcification, cord imaging, heart shadow enlargement, pleural effusion.	Acute	Rib fractures	–	Adults >16 years
Qure.AI	qXR	Radiography	30 findings including lung nodules, pneumothorax, pleural effusions, rib fractures, and pneumoperitoneum	Acute	Rib fractures only	–	Age not specified, presumed only adults
Qure.AI	qMSK	Radiography	Bone fractures and joint dislocations	Acute	Wrist, hand, finger, fibula and tibia, ankle, foot, shoulder, ribs, forearm	–	Age not specified, presumed only adults
SenseTime	SenseCare Lung CT	CT	Pulmonary nodules, pneumonia (including COVID-19) lesions and fractures	Acute	Rib fractures	–	Age not specified, presumed only adults
Infervision	InferRead CT Rapid Triage v1	CT	Coronary artery stenosis, chest fracture, ICH	Acute	Rib fractures	–	Age not specified, presumed only adults
Qure.AI	qER	CT	11 findings, including cranial fractures	Acute	Skull only	–	Adults
Aidoc	Briefcase for C-Spine Fracture triage (CSF)	CT	Cervical spine fractures	Acute	–	–	Age not specified, presumed only adults
Aidoc	BriefCase for Rib Fracture (RibFx)	CT	Rib fractures	Acute	–	–	Age not specified, presumed only adults
Nanox.AI	Bone Health Solution/HealthVCF	CT	Vertebral compression fractures	Acute and healing	–	–	Adults intended (>50 years)
Shanghai United Imaging Intelligence	uAI EasyTriage-Rib	CT	Rib fractures (detects when there are 3 or more fractures, not fewer)	Acute	–	–	Age not specified, presumed only adults

Abbreviations: MRI = magnetic resonance imaging, CT = computerized tomography, where available, the latest version of the AI product is provided.

**Table 3.** Licencing and user details of commercial AI tools for fracture detection on medical imaging.

Company	Product	CE certification	CE class	FDA certification	FDA class	FDA clearance date	Number of claimed users	Pricing strategy
Gleamer	BoneView 1.1-US	–	–	510(k)	2	January 31, 2023	Unknown	Annual or multi-year subscription (number of users, number of installations, number of analyses)
Gleamer	BoneView v2.0.2^a^	MDD *(MDR pending)*	2a	510(k) *(Adult only)*	2	March 1, 2022	>550	Annual or multi-year subscription (number of users, number of installations, number of analyses)
Radiobotics	RBfracture v.1	MDR	2a	–			>10	Pay-per-use, subscription, one-time license fee (number of analyses)
AZmed	Rayvolve v2.5.0	MDR	2a	510(k) *(Adult only)*	2	June 2, 2022	>700	Fixed-price annual subscription based on patient volumetry (trauma examinations per year). Free trial phase available
Rayscape	Chest X-ray	MDD	1	–			>100	Subscription (number of analyses)
Milvue	SmartUrgences	MDR	2a	–			>10	Subscription (number of users, number of installations, number of analyses)
Imagen Technologies	OsteoDetect	–		510(k) *(Adult only)*	2	May 24, 2018	Unknown	Unknown
Imagen Technologies	FractureDetect	–		510(k) (*Adult only)*	2	July 30, 2020	Unknown	Unknown
Annalise.AI	Annalise Enterprise CXR v1.2	MDR	2b	510(k) *(Pneumothorax only)*	2	February 24, 2022	>300	Subscription (number of analyses)
Quibim	Chest X-Ray Classifier	MDD	2a	–			Unknown	Licence (number of installations, number of analyses)
SenseTime	SenseCare-Chest DR Pro	MDR	2b	–			Unknown	Subscription, pay-per-use (number of users, number of installations, number of analyses)
Infervision	InferRead DR Chest v1.0.1.1	MDD	2a	–	–		Unknown	Subscription (number of installations)
Qure.ai	qXR	MDR	2b	510(k) *(Breathing tubes only)*	2	November 22, 2021	>1000 *(across all products)*	Pay-per-use
Qure.ai	qMSK	MDR	2b	–	–	–	>1000 *(across all products)*	Pay-per-use
SenseTime	SenseCare Lung CT	MDR	2b	–	–	–	Unknown	Subscription, pay-per-use (number of users, number of installations, number of analyses)
Qure.ai	qER	MDR	2b	510(k)	2	June 11, 2020	>1000 *(across all products)*	Pay-per-use
Aidoc	Briefcase for C-Spine Fracture triage (CSF)	MDD	1	510(k)	2	May 31, 2019	>500	Subscription (total imaging volume)
Aidoc	BriefCase for Rib Fracture (RibFx)	MDD	1	510(k)	2	April 14, 2021	>500	Subscription (total imaging volume)
Nanox.AI	Bone Health Solution/HealthVCF	MDD	2a	510(k)	2	May 12, 2020	Unknown	Subscription (£38 000 to £90 000 p.a.—number of analyses)
Shanghai United Imaging Intelligence	uAI EasyTriage-Rib	MDD	2a	510(k)	2	January 15, 2021	Unknown	Unknown

Abbreviations: MDD = Medical Devices Directive (pre May 26, 2021), MDR = Medical Devices Regulation (post May 26, 2021).

Deepnoid’s Deep: Spine-CF-01 only has approval from the Korean Ministry of Food and Drug Safety (No. 19-550).

a Previous version of the software from the company.

**Table 4.** Evidence for AI performance, based on FDA/CE conformity documentation or vendor endorsed studies.

Company	Product	Modality	Type of evidence	Predicate device (FDA)	Single/multicentre data	Readers/data set	Summary of evidence	Level of evidence	Ref.
Gleamer	BoneView 1.1-US	Radiography	FDA approval documentation	BoneView 1.0-US	Multicentre	2000 paediatric radiographs	High-sensitivity operating point: sensitivity 0.909 [95% CI, 0.889-0.926]. High-specificity operating point: specificity 0.965 [95% CI, 0.952-0.976].	Level 2	15
Gleamer	BoneView 1.1-US	Radiography	FDA approval documentation	BoneView 1.0-US	Multicentre	8918 adult radiographs	High-sensitivity operating point: sensitivity 0.928 [95% CI, 0.919-0.936] High-specificity operating point: specificity 0.932 [95% CI, 0.925-0.939]	Level 2	15
Gleamer	BoneView 1.1-US	Radiography	FDA approval documentation	BoneView 1.0-US	MCMR	480 cases, 14 clinical researchers	Reader specificity improved from 0.906 (95% CI, 0.898-0.913) to 0.956 (95% CI, 0.951-0.960). Reader sensitivity improved from 0.648 (95% CI, 0.640-0.656) to 0.752 (95% CI, 0.745-0.759).	Level 3	15,16
Gleamer	BoneView v2.0.2^a^	Radiography	FDA approval documentation	Imagen Technologies—FractureDetect	–	24 readers 480 examinations	Specificity improved from 0.906 to 0.956 Sensitivity improved from 0.648 to 0.752	Level 3	17
Radiobotics	RBfracture v.1	Radiography	Vendor conducted study	–	Multicentre (United States and Denmark)	8 readers 312 examinations	Sensitivity improved by 5.6% 47% reduction in false negative findings. AUROC was 0.97	Level 3	18
Azmed	Rayvolve v2.5.0	Radiography	FDA approval documentation	Imagen Technologies—FractureDetect	–	24 readers 186 examinations	Sensitivity improved from 0.86561 to 0.9554; Specificity improved from 0.82645 to 0.83116; AUROC improved from 0.84602 to 0.89327	Level 3	19
Rayscape	Chest X-ray	Radiography	Medical white paper by vendor	–	–	–	AUROC of 90.2.	Level 2	20
Milvue	SmartUrgences	Radiography	Medical white paper by vendor	–	Multicentre	8 readers 650 examinations	Sensitivity of junior radiologist with AI improved from 92% to 95%; Sensitivity of senior radiologists with AI improved from 93% to 95%	Level 3	21,22
Imagen Technologies	OsteoDetect	Radiography	FDA approval documentation	Not stated	Multicentre	24 readers 200 examinations	AUROC improved from 0.840 to 0.889; Sensitivity improved from 0.747 to 0.803; Specificity improved from 0.889 to 0.914.	Level 3	23
Imagen Technologies	FractureDetect	Radiography	FDA approval documentation	Imagen Technologies—OsteoDetect		11 970 examinations for diagnostic accuracy 24 readers, 175 examinations for MRMC study	Sensitivity 0.951 (95% CI, 0.940-0.960), Specificity 0.893 (95% CI, 0.886-0.898), AUROC 0.982 (95% Bootstrap CI, 0.9790, 0.9850). AUROC improved from 0.912 to 0.952; Sensitivity improved from 0.819 to 0.900; Specificity improved from 0.890 to 0.918	Level 3	24
Imagen Technologies	FractureDetect	Radiography	Vendor conducted study	–	Multicentre (United States)	24 clinicians, 175 cases	Improved AUROC from 0.90 [95% CI, 0.89-0.92] to 0.94 [95% CI, 0.93-0.95]. Improved sensitivity from 82% [95% CI, 79-84] to 90% [95% CI, 88-92]. Improved specificity from 89% [95% CI, 88-90] to 92% [95% CI, 91-93]	Level 3	25
Annalise.AI	Annalise Enterprise CXR v1.2	Radiography	Vendor conducted study	–	–	20 readers 4568 examinations in 2568 patients	AUROC score of 0.713 improved to 0.808 with AI.	Level 3	26
Deepnoid	DEEP: SPINE-CF-01	Radiography and MRI	Vendor endorsed study	–	–	160 radiographs	Dice 91.60% Sensitivity 90.12% Specificity 99.59% Precision 84.57%	Level 2	27
Qure.AI	qER	CT	FDA approval documentation	Aidoc’s Briefcase Software	Multicentre (United States)	1320 CT scans	Cranial fracture sensitivity of 96.77%, specificity of 92.72%, and AUROC of 0.9766	Level 2	28
Qure.AI	qER	CT	Vendor presentation	–	–	2971 scans	Per-scan image only AUROC was 0.72 and image with haemorrhage feature AUROC was 0.83.	Level 2	29
Qure.AI	qER	CT	Vendor poster	–	–	18 200 scans	Images only AUROC was 0.9599 and AP 0.7952 Images with scalp hematoma indicator AUROC was 0.9666 and AP 0.8190.	Level 2	30
Aidoc	Briefcase for C-Spine Fracture triage (CSF)	CT	FDA approval documentation	Aidoc Briefcase for ICH triage	Multicentre (3 sites)	186 examinations	Sensitivity of 91.7% Specificity of 88.6%	Level 2	16
Aidoc	BriefCase for Rib Fracture (RibFx)	CT	FDA approval documentation	Aidoc Briefcase for PE triage	Multicentre (3 sites)	279 examinations	Sensitivity of 96.7% Specificity of 90.4% AUROC of 0.976	Level 2	31,32
Nanox.AI	Bone Health Solution/HealthVCF	CT	FDA approval documentation	cmTriage	Multicentre (United States and Israel)	611 examinations	AUROC of 0.9504 (95% CI, 0.9348-0.9660) Sensitivity of 90.2% (95% CI, 86.3-93.05) Specificity of 86.89% (95% CI, 82.63-90.22) Average performance time was 61.36 seconds.	Level 3	33
Nanox.AI	HealthVCF	CT	NICE review (includes vendor funded study)				Specificity of 94% Sensitivity of 59% and Major osteoporotic fractures: 66.5% sensitivity 64.7% specificity Hip fractures: 92.6% sensitivity 36.9% specificity	Level 2	34
Shanghai United Imaging Intelligence	uAI EasyTriage-Rib	CT	FDA approval documentation	NanoxAI, HealthVCF	Multicentre	200 examinations	Sensitivity of 92.7% Specificity of 84.7% AUROC of 0.939. Time to notification of all 76 positive findings in study were 69.56 seconds.	Level 3	35

Preferential evidence provided where MRMC (multireader, multicase) studies were conducted to evaluate clinical improvement (rather than standalone bench testing results). A hyphen denotes either unknown/not stated or not applicable information. Although the product for Annalise.ai does have FDA approval, this is only for detection of pneumothoraces rather than fractures, therefore, the FDA clearance evidence is not included below. Similarly, Qure.AI has FDA approval for their qXR product for “breathing tube placement” analysis only, therefore, the FDA clearance evidence is not included below.

a Previous version of the software from the company.

**Table 5.** Evidence for AI performance, based on independent external peer reviewed publications.

Company	Product	Modality	Type of evidence	Readers/data set	Summary of evidence	Level of evidence	Ref.
Gleamer	BoneView v2.0.2^a^	Radiography	Retrospective study	500 patients, 3 radiologists	Sensitivity increased by 20% and specificity increased by 0.6%. PPV increased by 2.9% and the NPV by 10%. AUROC increased by 10.2%. Decreased mean reading time by 12.7 seconds.	Level 4	36
Gleamer	BoneView v2.0.2^a^	Radiography	Retrospective study	4774 radiographs	For fractures, dislocations, elbow effusions, and focal bone lesions, respectively: AI sensitivity higher by 24.4%, 26.6%, 6.8%, and 82%, specificity lower by 12%, 0.9%, 0.2%, and 4.4%.	Level 2	37
Gleamer	BoneView v2.0.2^a^	Radiography	Retrospective MRMC study	480 examinations, 24 readers	Per-patient sensitivity increased from 64.9% to 75.2%, specificity increased from 90.6% to 95.6%, decreased mean reading time by 6.3 seconds.	Level 4	38
Gleamer	BoneView v2.0.2^a^	Radiography	Retrospective MRMC study	600 patients, 6 radiologists and 6 emergency physicians	AI assistance improved sensitivity of physicians by 8.7%, specificity by 4.1%, reduced mean number of false positives in fracture diagnosis per-patient by 41.9% and reduced mean reading time by 15.0%. The stand-alone AI performance was better than all unaided readers with an AUROC of 0.94.	Level 4	39
Gleamer	BoneView v2.0.2^a^	Radiography	Retrospective study	300 radiographs, 3 senior paediatric radiologists and 5 resident radiologists	Using AI assistance, sensitivity for junior radiologists increased by 10.3%, senior radiologists by 8.2%. Junior radiologist specificity increased by 1.4% and senior radiologist specificity decrease by 0.2%. AI stand-alone sensitivity and specificity were 91% and 90%, respectively.	Level 3	40
Gleamer	BoneView v2.0.2^a^	Radiography	Retrospective study	1163 examinations, 2 resident radiologists	Radiologist unaided sensitivity was 84.74%, and AI algorithm stand-alone sensitivity was 86.92%. AI assistance increased sensitivity by 6.54% and specificity by 0.26%.	Level 3	41
Gleamer	BoneView v2.0.2^a^	Radiography	Retrospective study	1917 radiographs, 41 radiologists	Stand-alone AI sensitivity was 7% higher, specificity was the same. AI assistance increased sensitivity by 12% and decreased specificity by 4%.	Level 3	42
Gleamer	BoneView v2.0.2^a^	Radiography	Retrospective study	300 radiographs, 2 radiologists	Per-patient sensitivity across all fractures was 91.3% and specificity was 90.0%. The AUROC for all fractures was 0.93.	Level 2	43
AZmed	Rayvolve v2.5.0	Radiography	External validation study	5865 radiographs	95.7% sensitivity; 91.2% specificity; 92.6% accuracy.	Level 2	44
Milvue	SmartUrgences	Radiography	External validation study	300 radiographs, 26 radiologists	Accuracy was 79.5%, sensitivity as 83.6%, specificity was 75.2%.	Level 2	45
Annalise.AI	Annalise Enterprise CXR v1.2	Radiography	External MRMC study	2972 cases, 11 readers	Using AI assistance 92 cases (3.1%) had significant report changes, 43 cases (1.4%) had changed patient management and 29 cases (1.0%) resulted in further imaging recommendations.	Level 4	46
Annalise.AI	Annalise Enterprise CXR v1.2	Radiography	External validation study	1404 cases, 2 radiology residents	Radiologists performed better than AI for clavicle fracture (*P* = .002), humerus fracture (*P* < .0015) and scapula fracture (*P* = .014), no statistical difference for rib fractures.	Level 2	47
Qure.ai	qXR	Radiography	Prospective multicentre study	65 604 radiographs	AI rib fracture AUROC was 0.98 and NPV was 99.9%. Turnaround time decreased by 40.63% using AI.	Level 4	48
Qure.ai	qXR	Radiography	Retrospective multicentre study	279 cases	Rib fracture sensitivity was 87%, specificity was 100%, and accuracy was 94% in these cases that were previously initially missed or mislabelled in radiology reports.	Level 2	49
Qure.ai	qXR	Radiography	Retrospective study	127 cases, 5 radiologists	AI sensitivity was 26.4% higher. AI specificity was 15.9% lower.	Level 2	50
Aidoc	C-Spine (CSF)	CT	External validation study	665 examinations, (radiologists of different levels of expertise and training)	CNN accuracy lower than radiologist (92% [95% CI, 90-94] vs 95% [95% CI, 94-97]). CNN sensitivity lower than radiologist (76% [95% CI, 68-83] vs 93% [95% CI, 88-97]). CNN specificity higher than radiologist (97% [95% CI, 95-98] vs 96% [95% CI, 94-98]).	Level 2	51
Aidoc	C-Spine (CSF)	CT	External validation study	1904 cases, 1 attending neuroradiologist	AI and radiologist interpretation concordant in 91.5% of cases. AI correctly identified 54.9% of cases with 106 false positives. AI sensitivity was 54.9% [95% CI, 45.7-63.9], specificity was 94.1% [95% CI, 92.9-95.1], PPV was 38.7% [95% CI, 33.1-44.7], and NPV was 96.8% [95% CI, 96.2-97.4].	Level 2	52
Shanghai United Imaging Intelligence	uAI EasyTriage-Rib	CT	External validation study	393 cases	Per-patient level, AI set to detect all rib trauma—sensitivity was 90.91%, specificity was 76.21%, PPV was 77.63%, and NPV was 90.23%; AI set to detect displaced rib fractures—sensitivity was 95.56%, specificity was 74.59%, PPV was 52.76%, and NPV value was 98.26%.	Level 2	53

Abbreviation: MRMC = multireader, multi case study.

a Previous version of the software from the company.

The majority of the commercial AI products (14/21) were intended for fracture detection on plain radiographs (Figures 1–3), with the remainder (7/21) related to CT evaluation. Only 3 products specified they were intended for use in adults and children (all relating to radiographic interpretation), with the remainder intended solely for adult use. All products were intended to aid human interpretation or triage, not for autonomous usage (at this stage of their development or regulation).

## Evidence levels

Predominantly, the AI products reviewed had evidence for their performance provided by the vendor for conformity certification, with 7 having independent, peer reviewed publications available (total publications = 18), with the greatest number originating from Gleamer (*n* = 8). The majority of the evidence levels for AI product performance were at Level 3 (ie, change in diagnosis with and without AI assistance) and with some products (eg, BoneView *Gleamer*,^36^^,^^38^^,^^39^ Annalise Enterprise CXR v1.2 *Annalise.AI*,^46^ Qure.AI *qXR*^48^) potentially demonstrating evidence at Level 4 (ie, demonstrating improvement in time for diagnosis, which could be argued may lead to swifter treatment or follow-up for the patient^38^). There was no evidence available to demonstrate a benefit in actual patient outcome (eg, reduced time for recovery, reduction in repeated hospital visits, etc.), nor any publications on the health economic cost savings. Only one external validation article specifically mentioned changes to patient management (for Annalise Enterprise CXR v1.2 *Annalise.AI*).^46^ A summary of the available evidence associated with each product is reviewed in Table 4.

The largest independently published study to demonstrate improvement in human diagnostic accuracy with AI for fracture detection, included 480 radiographs (60 radiographs across 8 body parts; 50% abnormal) across 24 readers (comprising radiologists, emergency physicians, orthopaedic surgeons, and other healthcare professionals).^38^ There was an overall improvement in sensitivity rates across all specialists with AI assistance of 10.4%, and shortened reading by 6.3 s per examination.

Only one of the AI products (HealthVCF, Nanox.Ai) was reviewed by the National Institute for Health and Care Excellence (NICE) in a Medtech innovation briefing document,^34^ based on a published conference abstract^57^ and one peer-reviewed article,^58^ regarding the use of AI for the assessment of vertebral compression fractures on CT imaging. Whilst the NICE experts accepted that there would be clear patient benefit from the detection of vertebral compression fractures, and that the evidence was promising, it was nonetheless limited; the only published article was funded by the company.

Externally conducted studies that verify the performance of AI algorithms based on CT input are severely lacking. Only 3 such studies were identified, of which 2 are for the same product (Aidoc *C-Spine [CSF]*^51^^,^^52^). The study that included the largest number of cases involved 1904 CT scans and the performance of the AI algorithm was assessed against interpretation of the scans by a single attending neuroradiologist. The AI and neuroradiologist had agreeing reports in 91.5% of all cases. The AI was able to correctly identify 67 of 122 fracture cases (54.9%) and returned 106 cases that were false positives. The sensitivity, specificity, positive predictive value (PPV), and negative predictive value (NPV) of the AI algorithm were 54.9% (95% CI, 45.7-63.9), 94.1% (95% CI, 92.9-95.1), 38.7% (95% CI, 33.1-44.7), and 96.8% (95% CI, 96.2-97.4), respectively. The researchers also analysed the misdiagnosed fractures, finding that cases of chronic fractures were overrepresented, suggesting the AI algorithm is not well-adapted to handle this presentation.^52^

### Evidence for usage in children

All AI products were intended for use in adults, with independent peer-reviewed evidence for accuracy of the product in children (and younger adults) available for 2 vendors (AZMed and Gleamer). In one study evaluating the performance of the Rayvolve product (AZMed),^44^ a retrospective review of 2634 radiographs across 2549 children (<18 years age) from one single French centre was performed. This demonstrated an overall sensitivity of 95.7%, specificity of 91.2%, and accuracy of 92.6% for presence/absence of a fracture (regardless of number and whether the fracture was correctly localized or not). There was some reduction in the accuracy of the product for children aged <4 years old and those within a cast. While there was a similar sensitivity of fracture detection in patients with cast compared to without cast of 95.3% and 93.9% (1.4% difference), respectively, the difference in specificity was significant at 30.0% and 89.5% (50.9% difference), respectively. The accuracy of fracture detection also decreased to 83.0% in the case of patients with casts, compared to 90.7% in patients without cast (a difference of 7.7%). Results in fracture detection from the 0-4 years and 5-18 years age subgroups also showed a difference with sensitivity of 90.5% (0-4 years), and 95.4% (5-18 years) (difference of 4.9%). The specificity did not demonstrate significant differences at 88.9% (0-4 years) and 88.8% (5-18 years), however, the accuracy did slightly decrease to 89.3% (0-4 years), compared to 90.7% (5-18 years) (difference of 1.4%).

Two publications evaluated the use of the BoneView product (Gleamer) in children and young adults^40^^,^^43^ using the same data set of 300 musculoskeletal radiographs (half with fractures, in patients aged 2-21 years old) across 5 body parts acquired from a United States-based data provider. In the first study,^43^ an external validation of the AI product alone was performed demonstrating sensitivity of 91.3%, specificity 90%, and patient-wise AUROC of 0.93. Avulsion fractures were noted to be challenging for the AI tool to detect (sensitivity per fracture of 72.7%). In the second publication,^40^ differences in radiologist performance before and after AI assistance were evaluated across 8 radiologists (5 radiologists in training and 3 qualified paediatric radiologists). Across all 8 radiologists, the mean sensitivity was 73.3% without AI; and increased by almost 10% (*P* < .001) to 82.8% with AI. There was a statistically significant improvement in sensitivity for radiologists in training (by 10.3% [*P* < .001]) compared to specialist paediatric radiologists (8.2% [*P* = .08]) demonstrating greater benefit for less experienced radiologists.

### Conformity certification

For medical devices to be commercialized in different countries, different types and levels of conformity certification are mandatory. These do not necessarily guarantee the safety or efficacy of a product, more that it has been assessed and found to meet a certain minimum requirement. A list of the certifications for various products in this review is listed in Table 3.

In Europe and Northern Ireland, “CE certification” is required, however, recent changes to this regulation were introduced for medical devices. Prior to May 26, 2021, medical devices were CE certified under the “Medical Devices Directive” (MDD), however since then new standards known as the “Medical Devices Regulation” (MDR) have come into play. The MDR introduces more stringent requirements for clinical evidence, safety, post-market surveillance, and responsibilities of Notified Bodies (ie, the organizations designated to assess conformity with the regulations).^59^ CE certified medical devices under MDD will therefore be required to re-certify under MDR before December 31, 2028 (for medium and lower risk medical devices)^60^; it is therefore important to know what CE certification a product has prior to purchase. In this review, 7/16 products have the more recent MDR CE certification, 6/16 have MDD CE certification, and 3 do not have current CE certification.

In Great Britain (ie, England, Wales, Scotland), the UKCA (UK Conformity Assessed) marking is a new regulatory marking that applied to medical devices following the end of the Brexit transition period (December 31, 2021),^61^^,^^62^ although devices with CE marking will continue to be recognized until June 30, 2023 after which UKCA marking will be required for product use. At present, there is no central list of UKCA marked products, however, it is also possible to contact the relevant regulatory authority for further information (eg, the MHRA for UKCA). Although we contacted both bodies, we did not receive a timely response regarding which AI assisted fracture detection tools had this marking.

In the United States, FDA approval is required for medical devices and generally follows what is known as the “premarket notification (510(k)) process” for low to moderate risk devices (ie, Class 1 or 2 devices, which apply to the devices covered in this review). This pathway allows a vendor to demonstrate that their device is “substantially equivalent” to a legally marketed device (known as a “predicate device”) already on the market and the vendor must include enough information (eg, intended use, comparison to predicate device, safety data) to prove that it can be marketed without requiring the more extensive “premarket approval” work-up (PMA).^63^ In this review, 11/16 products reported FDA certification.

## Discussion

Our market review highlighted a range of commercially available AI products for fracture detection across a variety of body parts and imaging modalities, with most for radiographic assessment and intended for an adult population. Relatively few products have published independent peer-reviewed evidence for their efficacy and diagnostic accuracy in children, although where tested, AI performance was found to be reduced for younger children.

For adults, there was a larger amount of peer-reviewed evidence across different body parts, with more studies evaluating the benefit of radiologists’ imaging interpretation with and without AI and changes in speed of reporting for some products. It is hard to assess the best performing product purely through sensitivity and specificity due to varying levels and quality of evidence available; however, it is evident that Gleamer’s BoneView is the most extensively externally validated product, whilst also reporting impressive sensitivities and specificities. Studies conducted on this product have also demonstrated reduced reading times of radiographs. This highlights possible future benefit for patients, especially if this leads to a faster referral for specialist care and treatment, although evidence demonstrating downstream improved patient outcomes and cost savings for a hospital department are yet to be evaluated (ie, Level 5 and 6 evidence).

It is important however to understand the type of conformity certification an AI product has prior to purchase, and we have tried to be as comprehensive yet concise as possible in our review of the market status. There have been some notable changes in the CE certification regulations and also those for sale in the UK market. Many products which have previously been awarded CE certification under MDD will require re-certification under MDR soon, and those wishing to use a product in the United Kingdom will need to check that their vendor has/will receive the UKCA certification in the near future.

Through conducting this investigation into commercially available tools that leverage AI to perform detection and diagnosis of fractures, we have been able derive some key conclusions about this market.

First is the divide in modality, between plain X-ray radiographs and CT scans. The latter constitutes a significantly smaller portion of the products available for fracture detection and all found in this analysis only target section(s) of the axial skeleton for fractures. All CT-based products also specifically target one type of fracture, either relating to the spine or ribs, whereas products based on radiographs often tend to include many different body parts and different pathologies. Whilst the results we provide in this review are for overall summary diagnostic accuracy rates, it is important for readers to review the listed references and FDA documentation, where available, if they wish to garner more detailed accuracy rates for specific fracture locations and types.

Second, the market for children is still significantly behind adults in terms of range of available products, with 4 of the 16 products (25%) stating their applicability to children. There are understandably significant technical challenges in adapting AI solutions to be effective in paediatrics due to the variability in bone structure, predominantly between the ages of 0-16 years.^5^ Development in this more specialized field is also slowed and restricted by legal issues relating to the collection of data for training the algorithms and more complex and difficult procedures for obtaining certification or approval from respective legal bodies.^64^

Third, the amount of independent external validation is also significantly lacking for many products, with many only having vendor conducted validation for purposes of achieving FDA approval or CE certification. Future work in this domain that independently verifies the performance of specific commercially available products would provide a much clearer basis to evaluate which product is best for a clinical/health institution. Of all the evidence discovered throughout this review, the highest level of evidence was Level 4 (according to the levels of evidence suggested by Fryback and Thornberry^13^). This means study into the deeper impact of these tools is severely lacking, given that evidence Levels 5 and 6 assess the effect on patient outcomes through changes to quality of life and societal impact based on an economic analysis. As interest in this field continues to grow, such assessments will be fundamental in determining the greater value these products are able to provide.

We acknowledge that our review has limitations due to the ever-increasing number of commercial AI products coming to market and newer versions of existing tools being developed. It is likely that by the time of publication we may not have included some very recent tools, recent conformity accreditation and evidence to support usage in particular situations, which were unavailable at time of our search. We did contact as many AI companies as possible, including those that advertised only prototype versions of their software, to ensure we captured emerging products as well as those already established. We also offered the AI companies an opportunity to let us know of any updates in development. Some AI companies did not engage or respond to our request for information within the timeframe provided, including the MHRA regarding details on products with UKCA certification.

## Conclusion

Overall, there is a scarcity of rigorous, independent evaluation of commercially available AI tools for fracture detection in adults and children, and some products will need to update their current conformity registration. The information in this article may help departmental and hospital leaders, as well as local AI champions, in understanding whether tools available are worth further investigation for their specific institution at this stage in their development.

## Supplementary Material

tzad005_Supplementary_Data
