# Correction to: Commercially available artificial intelligence tools for fracture detection: the evidence

**DOI:** 10.1093/bjro/tzae004

**Published:** 2024-02-22

This is a correction to: Cato Pauling, Baris Kanber, Owen J Arthurs, Susan C Shelmerdine, Commercially available artificial intelligence tools for fracture detection: the evidence, *BJR|Open*, Volume 6, Issue 1, January 2024, tzad005, https://doi.org/10.1093/bjro/tzad005

The originally published version of this manuscript required correction.

Table 5 included an entry for Qure.ai and a citation of reference 50, “Kvak D et al. Chest X-ray abnormality detection by using artificial intelligence: a single-site retrospective study of deep learning model performance. BioMedInformatics. 2023;3(1):82-101”.

Table 5 has been updated to remove this information.

Subsequent references and their in-text citations have been renumbered accordingly.

