# Supplementary material for: Commercially available artificial intelligence tools for fracture detection: the evidence
Source: BJR Open. 2023 Dec 12;6(1):tzad005. doi: 10.1093/bjro/tzad005 (PMC10860511; doi:10.1093/bjro/tzad005)
Supplement: tzad005_Supplementary_Data [file tzad005_supplementary_data.docx]

**Commercially Available Fracture Detection AI Tools**

**Market Survey Questionnaire**

| **What AI products do you offer that can detect bone fractures (and which imaging modality does this work with)?** *Please include the software name and version number, and include as many products as you think are relevant* |
| --- |
| Product name:  Version number:  Imaging modality:  Product name:  Version number:  Imaging modality: |
| **Is the tool capable of detecting both acute and healing fractures?**  *If you listed more than one tool above, please be clear which tool you are talking about* |
|  |
| **Which other pathologies (other than bone fractures) does the tool evaluate?**  *If you listed more than one tool above, please be clear which tool you are talking about* |
|  |
| **Which body part(s) is the tool designed to evaluate?**  *For example – is it relevant for chest, spine, limbs, or all body parts?*  *If you listed more than one tool above, please be clear which tool you are talking about* |
|  |
| **Are there any specific body parts excluded (e.g. axial skeleton, dental xrays)?**  *If you listed more than one tool above, please be clear which tool you are talking about* |
|  |
| **What is the intended target population for the tool?**  *(Please provide an age range e.g. ages 5 years old + ; or only adults aged 18 years + etc)* |
|  |
| **What product licencing does the AI tool currently have?**  *i.e. Is it licenced for use on adults +/- children? Any licences currently being applied for/pending outcome?* |
|  |
| **What is the approximate number of clinical institutions that currently use the tool? Where are they based?** *(e.g. Europe only, worldwide etc)* |
|  |
| **How is your pricing structure worked out?**  *(e.g. is it by number of users, images, licences, monthly/annual subscription etc)* |
|  |
| **What evidence is available to support the product’s performance and outcomes (including unpublished work)?**  *Please feel free to send us the articles/pdfs or state the reference below where relevant*. |
|  |
| **In our article we would like to include some images/figures of AI tools in use.**  **Would you be willing to share a screenshot or example image of your tool detecting an abnormality for us to use if we have space?**  *Please feel free to send us jpegs/tiff files and a caption of what the image is showing that we could potentially use if we have space in our article.* |
|  |
